# Primary Bone Tumors and Breast Cancer-Induced Bone Metastases: In Vivo Animal Models and New Alternative Approaches

**DOI:** 10.3390/biomedicines12112451

**Published:** 2024-10-25

**Authors:** Argia Ucci, Luca Giacchi, Nadia Rucci

**Affiliations:** Department of Biotechnological and Applied Clinical Sciences, University of L’Aquila, 67100 L’Aquila, Italy; argia.ucci@univaq.it (A.U.); luca.giacchi@student.univaq.it (L.G.)

**Keywords:** animal model, osteosarcoma, bone metastasis, organ on a chip, organoids

## Abstract

Bone is the preferential site of metastasis for the most common tumors, including breast cancer. On the other hand, osteosarcoma is the primary bone cancer that most commonly occurs and causes bone cancer-related deaths in children. Several treatment strategies have been developed so far, with little or no efficacy for patient survival and with the development of side effects. Therefore, there is an urgent need to develop more effective therapies for bone primary tumors and bone metastatic disease. This almost necessarily requires the use of in vivo animal models that better mimic human pathology and at the same time follow the ethical principles for the humane use of animal testing. In this review we aim to illustrate the main and more suitable in vivo strategies employed to model bone metastases and osteosarcoma. We will also take a look at the recent technologies implemented for a partial replacement of animal testing.

## 1. Introduction

### 1.1. Bone Physiology and Key Players in the “Virtuous Cycle”

Bone is a highly dynamic connective tissue, composed of resident cells (osteoprogenitor cells, osteoblasts, osteocytes and osteoclasts) and an extracellular matrix, which has the unique characteristic of being mineralized. The bone matrix undergoes continuous cycles of deposition and degradation throughout a human’s life, to change bone segments shape and size and to maintain skeletal mechanical properties. These cycles, named bone modeling (during growth) and bone remodeling (in adulthood), are both physiological processes finely regulated to ensure the concerted activity of osteoblasts, the bone forming cells, and of osteoclasts, the bone resorbing cells [1,2,3]. Both cytotypes act in a coordinated manner under physiological conditions.

**Figure 1:**
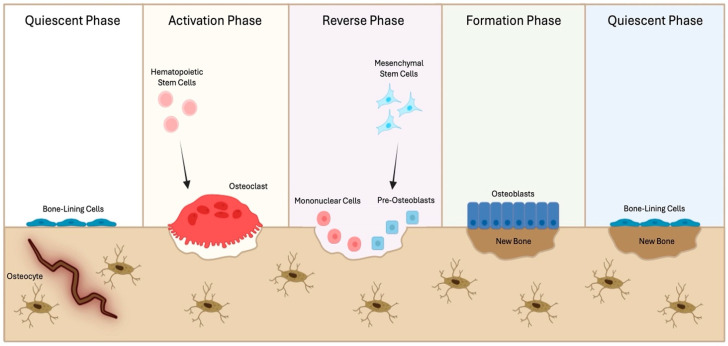
The “virtuous cycle” of bone remodeling. After stimuli of a different nature, bone-lining cells start producing factors which in turn recruit hematopoietic stem cells, inducing osteoclast differentiation and bone resorption (Activation phase); then macrophage-like cells (i.e., reverse cells) phagocyte the debris formed in the resorption area (Reverse phase), while growth factors released from the degraded bone matrix attract mesenchymal stem cells, promoting their differentiation in osteoblasts and the synthesis of new bone (Formation phase), thus closing the “virtuous cycle” of bone remodeling (Quiescent phase). Cartoon created with BioRender.com, accessed on 31 July 2024.

Bone remodeling is characterized by the following phases [4] (Figure 1):

Quiescent phase: stimuli such as microfractures and changes in mechanical loading perceived by osteocytes, or systemic/paracrine factors released in the bone microenvironment, activate the bone-lining cells, which cover the bone surface making it inaccessible to osteoclasts. Under local or systemic stimulation, these cells start producing factors which recruit osteoclast precursors and promote osteoclast differentiation.

Activation phase: osteoclasts resorb the bone matrix. This is a rapid phase, lasting about 2–3 weeks in humans. Once osteoclasts have completed the resorption process, they undergo apoptosis, activating the reverse phase.

Reverse phase: it couples bone resorption with bone formation and the name is due to the presence of macrophage-like cells, called reverse cells, which phagocyte debris releases after bone matrix degradation in the resorption area.

Formation phase: this is the longest phase and lasts 4–6 months. Growth factors usually stored in the bone matrix and released after its degradation attract osteoblast precursors and promote their differentiation in osteoblasts, which lay down an organic matrix (osteoid) and then mineralize it.

Several systemic and paracrine factors strictly regulate bone remodeling by influencing the activity of osteoclasts, osteoblasts, and osteocytes, keeping a balance between osteoclast-mediated bone resorption and osteoblast-mediated bone formation, in the so-called virtuous circle.

As for osteoclasts, hormones like parathyroid hormone (PTH), 1,25-dihydroxyvitamin D3, and thyroxine (T4) increase the production of receptor activators of the nuclear factor-kB ligand (RANKL) by bone marrow stromal cells and osteoblasts. RANKL binds to RANK receptor expressed on the osteoclast precursor surface and via the activation of the nuclear factor-kB (NF-kB) and c-Jun N-terminal kinase (JNK) pathways, to stimulate osteoclast formation and survival [5]. In addition, the release of Interleukin (IL)-6, IL-1β, prostaglandins, and colony-stimulating factors (CSFs) by osteoblasts induces the formation of osteoclasts and, consequently, increases bone resorption [6]. On the other hand, bone marrow resident cells can also produce cytokines that negatively regulate osteoclast formation, such as IL-4, IL-18, and Interferon (IFN)-γ [7].

Similarly, both paracrine and systemic factors finely modulate osteoblast proliferation, differentiation, and activity [8]. Among the former, the main regulators are those growth factors normally trapped in the bone matrix and released after the bone resorption process, including bone morphogenetic proteins (BMPs), transforming growth factor (TGF)-β, insulin-like growth factors (IGFs), and fibroblast growth factors (FGFs). Additionally, systemic factors that positively regulate bone formation include PTH, prostaglandins, and growth factors like platelet-derived growth factor (PDGF), while corticosteroids negatively regulate bone formation by inducing osteoblast apoptosis [9].

Finally, PTH, estrogens, 1,25-dihydroxyvitamin D3, irisin, calcitonin, TGF-β and inorganic phosphate (Pi) are key regulators of osteocytes lifespan, as well as network formation, secretome, and mechanotransduction properties [10]. They exert a direct effect on osteocytes inducing the release of local factors, such as sclerostin (SOST), dickkopf (DKK)-1, FGF-23, RANKL, osteoprotegerin (OPG), osteocalcin (OCN), dentin matrix acidic phosphoprotein (DMP)-1, phosphate-regulating endopeptidase homolog X-linked (Phex), matrix extracellular phosphoglycoprotein (MEPE), semaphorin (Sema)-3A, critically regulating the bone remodeling balance [11].

### 1.2. Primary Bone Tumors

Primary bone tumors (PBTs) are a group of malignancies originating from mesenchymal cells. These tumors represent less than 0.2% of overall cancer diagnosis but, unfortunately, 5% of overall childhood malignancies [12,13]. In childhood and adolescence, osteosarcoma presents with the highest incidence (56%), followed by Ewing sarcoma (ES) (34%), while in adults chondrosarcoma is the most frequent (40%), followed by osteosarcoma (28%) [14,15]. PBTs also include very rare subtypes, such as chordoma, undifferentiated pleomorphic sarcoma, adamantinoma, fibrosarcoma, and giant cell tumor of the bone. Each of them varies in demographics, imaging appearance, and biological behavior [13]. Also, sex seems to be a risk factor, since PBTs have a male predominance (male-to-female ratio: 1.43 to 1) [16,17].

#### 1.2.1. Osteosarcoma

As already stated, osteosarcoma is the most prevalent PBT, especially in adolescents and young adults [12,17]. The most common affected sites are the distal femur, proximal tibia, and proximal humerus. In adults, the axial skeleton is more commonly involved, where bone metabolic disease is often associated with this malignancy [18]. This tumor is highly aggressive and is divided into two main histological subtypes, called intramedullary and surface osteosarcoma, according to the tumor’s bone location and grade. The former can be further subcategorized as conventional (the most frequent, 80% of all osteosarcomas), telangiectatic (<4%), low–grade (<2%), and small–cells (1.5%); the surface subtype could be parosteal (1% to 6%), periosteal (1% to 2%), or high–grade osteosarcoma (<1%) [12,18].

Genetic changes are not present to explain the growth of this tumor; however, some inherited germline mutations have been associated with a predisposition for osteosarcoma. Indeed, there are eight currently known syndromes in which osteosarcoma occurs at an increased frequency: Li–Fraumeni syndrome (mutation of TP53 gene); retinoblastoma syndrome (mutation of RB1 gene); Rothmund–Thomson syndrome (mutation of RECQL4 gene); RAPADILINO syndrome (mutation of RECQL4 gene); Werner syndrome (mutation of WRN gene); Bloom anemia (mutation of RECQL3 gene); and Diamond–Blackfan anemia (mutation of S19 gene) [19].

From a biological and clinical point of view, an osteosarcoma is characterized by a disorganized bone structure, which is mainly arranged in irregular clumps of the osteoid matrix [20]. A diagnosis is based on several imaging approaches, including radiography, magnetic resonance imaging (MRI), computed tomography (CT), whole-body bone scintigraphy (Bone Scan), and positron emission tomography (PET), combined with laboratory tests and tissue biopsies [12,16]. To date, the treatment of osteosarcoma requires a multidisciplinary approach that includes a neoadjuvant multi–agent polychemotherapy (MAP) based on the administration of doxorubicin, cisplatin, and high-dose methotrexate [21,22], followed by a surgical excision and, eventually, by further adjuvant chemotherapy/radiotherapy cycles. The clinical outcome depends on the presence of metastatic foci and micrometastatic lesions, which mainly develop in the lungs, thus lowering the 5-year survival rate to 20% to 30% of cases [22].

#### 1.2.2. Chondrosarcoma

Chondrosarcoma is, among PBTs, the most frequently diagnosed in elderly people (40–70 years of age) [23] and can be further classified into primary, central when present in the medullary canal, and secondary peripheral if developing from the surface of the bone secondary to a pre-existing enchondroma/osteochondroma. Histologically, the grading of both subtypes ranges from grade I (or low grade), to grade II (intermediate grade), and grade III (or high grade) [24]. Usually, it occurs in the long bones of the appendicular skeleton; however the pelvis, ribs, and scapula can also be affected, although with less incidence.

Cytogenetic studies have highlighted chromosomal abnormalities, while gene mutations in Exostosin 1/2 (EXT1/2), TP53, Retinoblastoma 1 (RB1), and Isocitrate Dehydrogenase 1/2 (IDH1/2) have been associated with malignant transformation [16,25]. Histologically, chondrosarcoma has a cartilage-like appearance, being characterized by variously differentiated cells producing a chondroid matrix [26]. The diagnosis relies on the same approaches previously described for osteosarcoma, but the anatomical depth of these lesions often leads to a poor prognosis due to late diagnosis and high resistance of this tumor to chemotherapy and radiotherapy. Therefore, the only therapeutic option is represented by surgical resection [25,27].

#### 1.2.3. Ewing Sarcoma

Among all PBTs, Ewing sarcoma is the most aggressive. It is frequently diagnosed between 5 and 20 years of age, with a peak of incidence at 15 years and a male predominance of 1.5 to 1 for females [28]. The most recurrent affected sites are the diaphysis of long bones, pelvic bones, and the axial skeleton (ribs and vertebral column). Ewing sarcoma is genetically well described, with characteristic chromosomal translocations identified [28]. Translocations often lead to fusing a FET protein (EWSR1) to an ETS transcription factor (most commonly FLI1), thus resulting in the formation of an oncogenic fusion protein (EWSR1-FLI1) that induces transcriptional modification, activating the expression of genes such as the nuclear receptor subfamily 0, group B, member 1 (NR0B1), the enhancer of zeste (EZH2), the NK2 homeobox 2 (NKX2.2), lysine demethylase 3A (KDM3A), glioma-associated oncogene homolog 1 (GLI1), the Meis homeobox 1 (MEIS1), and the interleukin 1 receptor accessory protein (IL1RAP). On the other hand, EWSR1-FLI1 reduces the gene expression of the transforming growth factor beta receptor 2 (TGFBR2), the insulin like growth factor binding protein 3 (IGFBP3), lysyl oxidase (LOX), forkhead Box O1 (FOXO1), sprouty RTK signaling antagonist 1 (SPRY1), and p21 [29,30,31].

From a clinical point of view, Ewing sarcoma is characterized by rapid tumor growth and metastasis development (particularly in the lungs); the diagnosis is performed by imaging techniques, laboratory tests, and tissue biopsy [32]. The standard of care includes neoadjuvant chemotherapy, local treatment, and adjuvant chemotherapy [33]. The most frequent chemotherapy regimen used is known as VDC/IE and it alternates two different drugs combinations, given every 2 to 3 weeks: the first combination includes vincristine, doxorubicin, and cyclophosphamide; the second combination includes ifosfamide and etoposide [32]. Local treatment may involve surgical resection and/or radiation therapy [33]. The 5-year survival rate for localized Ewing sarcoma is 70–80%, but patient outcomes worsen in cases of pelvic involvement, large tumors, or incomplete tumor regression after chemotherapy [34].

### 1.3. Bone Metastases: How Tumor Cells Turn the “Virtuous Cycle” into a “Vicious Cycle”

Bone is one of the preferential sites of metastasis, with an incidence of 65% to 90% in prostate cancer, 65% to 75% in breast cancer, 17% to 64% in lung cancer, and 10% in colorectal cancer [35,36]. Once bone metastases occur, the chances of survival dramatically drop, as well as the quality of life, since affected patients usually experience intense bone pain, spinal cord compression, pathological fractures, and hypercalcemia, which can lead to kidney dysfunction, cardiac arrhythmias, and death [37]. Like other metastases, bone lesions are incurable and, so far, only palliative treatments are available, which may improve quality of life but not overall survival [36].

From a clinical point of view, bone metastatic lesions can be classified as osteolytic, osteosclerotic, and mixed. The former are the most frequent and typically observed in patients with breast cancer, while prostate cancer patients usually develop osteosclerotic (e.g., osteoblastic) bone metastases [38,39] characterized by the increased deposition of a poor quality and immature bone matrix. Osteolytic lesions represent areas of the skeleton (i.e., vertebrae, sternum, femur) where there is no more bone, which has been completely degraded by an exacerbated osteoclast activity fomented by tumor cells [38,39].

**Figure 2:**
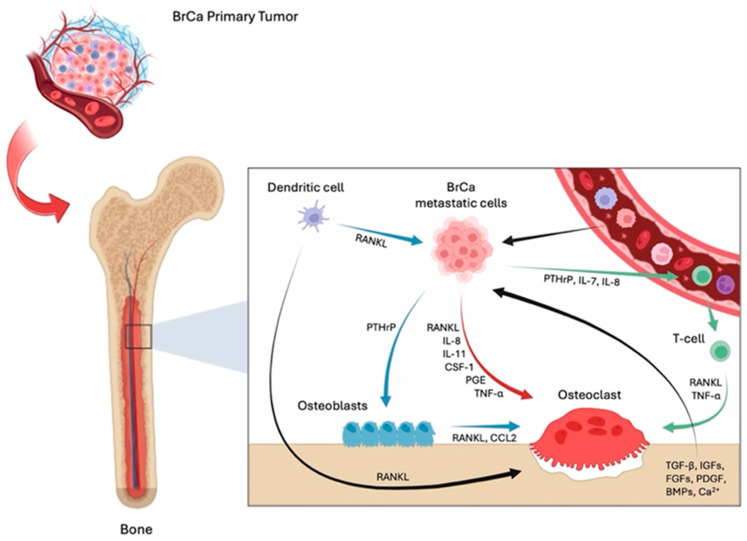
The “vicious cycle” of breast cancer-induced bone metastases. Cartoon illustrating the mechanisms by which osteolytic bone lesions develop. Breast cancer (BrCa) metastatic cell release factors that stimulate both indirectly (acting on osteoblasts) and directly on osteoclast formation, eventually leading to increased bone resorption. Osteoclastogenesis is also stimulated by immune cells, like dendritic and T-cells. Consequently, factors released from the bone matrix degradation feed tumor cells and create fertile soil for their growth and survival. Cartoon created with BioRender.com, accessed on 1 July 2024.

One of the first theories adopted to explain metastasis development came from Batson’s anatomical studies. This scientist proved that venous blood from the bladder, breast, and prostate flows not only in the system of hollow veins, but in a venous–vertebral plexus lacking a valvular system, extending from the pelvis (through the epidural and perivertebral veins) to the brain [40]. In addition to the hemodynamic theory, the “seed and soil” theory, proposed by Paget in 1889, underlined the importance of the host environment in the selectivity of cancer cells to metastasize to a target organ [41]. This process requires specific interactions between tumor cells (seed), the circulatory system, and the bone microenvironment (soil). Bone is also a storehouse of calcium and growth factors, which are released during bone resorption, thus feeding the “fertile soil” in which tumor cells can proliferate. Hence, as described by Roodman in 2004, when cancer cells reach the bone, they destroy the balance between osteoblasts and osteoclasts, thus resulting in a switch from a “virtuous cycle” to a “vicious cycle” [42] (Figure 2).

Intriguingly, breast cancer cells acquire an osteomimetic phenotype, producing osteoclastogenic cytokines such as CSF-1, PTHrP, RANKL, IL-8, IL-11, prostaglandin E, matrix metalloproteinase 1 (MMP-1), and TNF-α [43,44]. These factors directly lead to bone resorption by increasing osteoclast differentiation and activity. Consequently, the release of growth factors from the bone matrix perpetuates tumor growth [40,43].

The crosstalk between immune cells and the bone tumor microenvironment is also noteworthy. Indeed, it is known that dendritic cells are a source of RANKL, thus directly supporting osteoclastogenesis. RANKL produced by these cells can stimulate a tumor’s secretion of PTHrP [45], which in turn indirectly induces osteoclast formation by upregulating osteoblast production of RANKL [45,46,47] and C-C motif chemokine ligand 2 (CCL2) [48]. Conversely, tumor cells produce PTHrP, IL-7 and IL-8, that recruit T cells which, in turn, secrete TNF-α and RANKL, thus fomenting osteolytic bone metastases [49]. Also, macrophages promote breast cancer-induced bone metastasis through the IL-4 receptor (IL-4R), while monocyte/macrophage-restricted IL-4R ablation reduces the occurrence of this disease [50]. Moreover, myeloid-derived suppressor cells (MDSCs) produce nitric oxide (NO) which mediates the immunosuppression of T cells, and osteoclast generation directly affects MDSCs by inducing their differentiation into osteoclasts [51]. Therefore, breast cancer cells are not only able to reprogram the bone microenvironment by directly acting on osteoclasts and osteoblasts, but by influencing the activity of the immune cells, which concur to perpetuate the vicious cycle.

## 2. Animal Models in Bone Oncology

Proven to be indispensable for preclinical cancer research, animal models play a crucial role in understanding the pathogenesis and the molecular mechanisms of diseases, as well as in evaluating potential treatments to improve clinical outcomes. As a matter of fact, the choice of the appropriate animal model is critical for advances in bone cancer research.

In the following paragraphs, we will explore the animal models of osteosarcoma and breast cancer-induced bone metastases and their recent developments, highlighting their contributions to preclinical cancer research. Moreover, some ethical considerations will be addressed, with the aim of guaranteeing, as much as possible, animal welfare. In this regard, the recently published Oncology Best-practices: Signs, Endpoints and Refinements for in Vivo Experiments (OBSERVE) guidelines offers a comprehensive overview of the recommendations on refinements applied to murine cancer models [52].

### 2.1. Animal Models of Bone Primary Tumors

Primary bone cancers can be reproduced using various animal models essential to study tumor initiation, including progression, the ability to induce metastasis to the lungs, and response to treatments. Especially for osteosarcoma, several animal models deriving from different species are available, including dogs, mice, rats, and zebrafish [53,54], while according to the procedure employed, the types can be distinguished as follows: (1) spontaneous models, (2) cancer cell line injection models, (3) genetically engineered models, and (4) chemically induced models (Table 1, Table 2, Table 3 and Table 4). Each model has its strengths and limitations, as will be addressed in the following paragraphs.

In addition to cancer cells, fresh tumor fragments can also be transplanted. This in vivo model is referred to as a patient-derived xenograft (PDX), which consists of transplanting pieces of bone tumor directly collected from human patients into an immunodeficient host. Despite the limitations due to the ethical documentation required and availability of fresh human tissue, PDXs have been increasingly used in osteosarcoma and Ewing sarcoma research, for molecular, genetic and therapeutic investigations [55,56,57]. Interestingly, cell lines can also be generated from PDX models and are easy to cryopreserve and store for later applications [58].

#### 2.1.1. Spontaneous Models

Spontaneous models most commonly involve dogs, which could spontaneously develop osteosarcoma. Canine osteosarcoma is similar to the human disease at the histopathological and genetic level, thus representing a useful comparative tumor model [59,60,61]. Indeed, some of the genes involved in human osteosarcoma pathogenesis, such as P53 and RB, also appear to be involved in the development of canine osteosarcoma. However, there are some limitations to be considered in this animal model. First, osteosarcoma affects skeletally mature bone, and it mostly occurs in middle-aged and old dogs (peak of incidence 11–12 years of age) [62], which is a different age of development compared to humans, where the peak of incidence occurs during childhood and adolescence. Second, the tumor localization and incidence of the malignant disease are different, as canine osteosarcoma occurs preferentially in the distal radius and proximal humerus. Currently, the spontaneous osteosarcoma model is rarely used, due to the prolonged period needed for tumor development and to the heterogeneity of the tumor, which makes it difficult to conduct comparative studies [63,64]. Finally, it should be noted that dogs are companion animals, and their treatment depends on the owner’s decision.

#### 2.1.2. Cancer Cell Line Injection or Tissue Fragment Transplantation Models: Syngeneic Versus Xenograft Cancer Models

Widespread models involve implanting cancer cells or tumoral tissue fragments directly into mice or rats. These models can recapitulate key aspects of primary bone cancer, including tumor growth, invasion of surrounding tissues, and metastasis to lungs. Xenograft of human tumor cells, which requires the employment of immunocompromised mice, is the most widely used in vivo models in current oncological research and is used to identify factors involved in tumor migration and, most importantly, for antitumorigenic drug screening. On the other hand, allograft models in immunocompetent mice allow us to study not only tumor growth and metastatic potential, but the role of the immune system in controlling primary bone tumors and drug responses. Both approaches share the limitation of using fully developed cancer cells, which could not provide information about the initiation of the tumor and its etiology. There are numerous studies related to the development and use of xenograft and allograft models of human and murine-derived osteosarcoma cell lines injected into mice [65]. For Ewing sarcoma, established cell lines and patient-derived primary ES tumors harboring the EWS–FLI1 fusion are used to grow in vivo ES xenograft tumors [66]. However, these last xenograft models do not form metastatic foci. Recently, a humanized orthotopic mouse model has been developed for preclinical evaluation of immunotherapy in Ewing sarcoma [67], as will be described later.

It is important to consider that biologically different transplanted cells, mouse strains, and different sites of inoculation are all factors that can influence the OS formation rate, lung metastasis rate, and chemosensitivity [53,54,68]. The most used injection sites for cancer cell implantations are orthotopic, intraosseous, subcutaneous, and tail veins. Understanding the benefits and limitations of each model is critical to designing the appropriate experiment.

Whatever the type of tumor cells to be injected, these should be employed at a confluence less than 80%; moreover, any tumor cell contamination with mycoplasma or any other pathogen needs to be checked, to guarantee the reliability of the results and, most of all, the wellness of the recipient mice.

##### Subcutaneous Injection

The subcutaneous model is considered heterotopic, because the OS cell injection site is different from the one of origin. This model has distinct advantages and is often used by researchers, especially to screen drugs, thanks to the easy and direct accessibility of the resulting tumor; moreover, according to the OBSERVE guidelines, this procedure is classified as mild or moderate, the latter in case the implanted tumor develops ulceration [52]. This model is characterized by a high rate of incidence and reproducibility, as well as by a simple and non-invasive execution. Indeed, all injected mice usually develop the tumor, mortality following inoculation is zero and, if it occurs, is generally due to a side effect of the anesthesia; the site of inoculation, that is the dorsum, is also well tolerated by mice because it does not interfere with deambulation or breathing. It is also acceptable to inject cells on both flanks, which would allow them to reduce the number of mice. To further facilitate cell engraftment, OS cancer cells can be also incorporated into active bio-molecule scaffolds, such as a Matrigel based–matrix [69]. Finally, subcutaneously injected mice do not develop cachexia, a paraneoplastic syndrome characterized by high morbidity, eventually leading to death, as will be discussed in more detail (Section 2.3.1). On the other hand, important limitations to this model are the inability to fully recapitulate the appropriate natural tumor microenvironment and the fact that metastases are rarely observed, probably due to the slower growth rates of tumors grown subcutaneously. Usually, the duration of the experiment is 3–4 weeks, and it depends on the ability of tumor cells to grow; however tumor volume should not exceed 2 cm^3^ for mice and 4 cm^3^ for rats.

##### Tail Vein Injection

Metastatic lesions may be better studied by injecting OS cells directly into the tail vein of the murine models, under anesthesia, to investigate their capability to extravasate from vessels and proliferate in distant organs. This method often results in the development of tumor nodules in the lungs of osteosarcoma animal models [70,71]. However, injecting tumor cells directly into the circulation does not allow us to study the prerequisite steps of metastasis, prior to invasion of the intravascular space by tumor cells. The procedure is quite challenging compared to other methods of injection and usually the tail is preheated at 37 °C to promote vasodilation. The incidence of developed metastasis is high, but affected mice suffer from a debilitating condition, which could be managed by analgesic treatment or, if conditions worsen, to euthanasia, after a veterinary consultation. Usually, the duration of the experiment is around 4 weeks, and this procedure is classified as severe.

Although detrimental for the animals, this technique could be noteworthy if employed, if we consider that metastasis occurrence is the leading cause of a patient’s death.

##### Orthotopic Injection

This technique consists of the inoculation of osteosarcoma cells into the bone, that is the site of tumor development. It is widely used, thanks to the rapid tumor growth and high metastasis rate, although the site of injection could be more challenging than the subcutaneous one. Two types of orthotopic models can be distinguished as follows: the paratibial and the intratibial inoculation. Both models are likely to induce pain, therefore analgesia should be provided. A stressful condition is also due to the subsequent development of lung metastases; therefore, these procedures should be classified as severe. Usually, the duration of the experiment is no more than 4–5 weeks.

**Figure 3:**
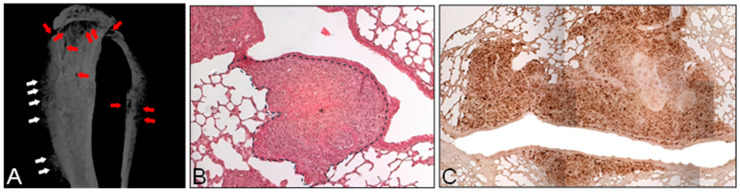
Orthotopic injection of human osteosarcoma cells. Four-week-old Balb-c nude male mice were anesthetized and subjected to paratibial injection (i.e., intramuscular inoculation) of 1 × 10^6^ human osteosarcoma MNNG/HOS cells. After 3 weeks the mouse was sacrificed, hindlimbs and lungs were explanted and subjected to ex vivo analysis. (**A**) Three-dimensional reconstruction of the paratibially injected tibia by ex vivo microCT scanning. Red arrows = area of osteolysis, white arrows = area of ectopic bone. (**B**,**C**) Histological sections obtained from the lungs of a paratibially injected mouse, showing micrometastases that have infiltrated the lung parenchyma, stained with (**B**) hematoxylin and eosin or subjected to (**C**) immunohistochemistry for the nuclear proliferative marker ki67. Dot line and asterisks = tumor; Scale bar = 100 μm.

In the paratibial model, tumor cells are injected into the caudal gastrocnemius muscle, close to the periosteum, allowing cells to expand into the adjacent bone during progression, to migrate into the vasculature, and to seed in distant sites like the lungs. The advantage of this model is that it more accurately recapitulates primary tumor growth and lung metastasis development [72,73]. Moreover, primary tumor growth is present with the area of ectopic bone deposition along with osteolytic lesions (Figure 3). In a recent study conducted by Crenn et al., paratibial injection of 3 × 10^6^ MOS-J cells in C57BL/6J mice was compared to subcutaneous and intraosseous syngeneic models, showing a tumor growth (volume) higher than the tumor induced by subcutaneous injection, and developing periosteal damage and cortical sclerosis [74].

The intratibial model consists of injection of cancer cells directly into the bone/bone marrow, typically into the proximal tibia [75,76,77].

Although the orthotopic model remains the best option, it may present a certain degree of variability in the efficiency of bone tumor establishment, growth, and ability to metastasize. Moreover, both types of injection require technical skills and a low volume of injection, since a larger amount of inoculum can lead to a deposition of tumor cells into the adjacent soft tissue, such as the surrounding musculature or the bone marrow, this last causing immediate metastasis, independent of development of a primary tumor. However, this model likely allows for a deeper and wider investigation of the osteosarcoma disease, which includes primary growth in the orthotopic site, blood vessel colonization, extravasation, homing, and colonization of the lung, thus recapitulating metastatic progression from the primary site [77]. Moreover, although both types of injections could be trickier than the subcutaneous one, usually the rate of success is close to 100% in terms of intraosseous growth of OS cells and almost 0% mortality. With regards to the development of lung metastases, this is linked to the aggressiveness of the OS cells injected.

##### Humanized Orthotopic Mouse Models

These models have been extensively studied over the past three decades, with numerous detailed reviews published [78,79,80,81] following the first humanized mouse models reported in 1988. Currently, different humanized mouse models are used in cancer research, providing a more accurate representation of human physiology and pathology. According to the types of human-derived cells or tissues transplanted, humanized mouse models can be classified into four main types, each offering distinct advantages and limitations to be carefully considered for experimental studies.

The SCID-Hu model is the first humanized mouse model developed. It is obtained by injecting mice with human CD34+ embryonic liver cells and transplanting human embryonic thymus and lymph nodes. Besides the obvious ethical implications, this model has stability issues with human-derived T cell development and a short lifespan, limiting its use to studying the pathogenesis of HIV [82].

The Hu-PBL (peripheral blood leukocyte)-SCID model has been obtained by injecting mature human peripheral blood leukocytes into adult SCID mice intraperitoneally or via the tail vein. After one week, low levels of human immune cells are detected, but the reconstitution of human-derived lymphocytes is often unstable. Injections of a larger number of cells can lead to complications such as Epstein–Barr virus-related B-cell lymphoma and significant allograft rejection, often resulting in short experimental windows (usually a couple of weeks after PBL injection) due to GVHD. In terms of costs and timing, it is the most convenient model used in T-cell-related immune research.

The Hu-hematopoietic stem cell (HSC) model is performed by injecting human CD34+ obtained from umbilical cord blood, via the tail vein or bone marrow cavity of immunodeficient mice, pre-irradiated with sublethal γ-irradiation to favor the HSC engraftment. The model can be set up in two ways, in adult or neonatal mice, with differences in the outcome regarding the production of human immune cells. Despite the fact that cell differentiation takes a minimum of 10 weeks, it is widely used in studying immune responses and viral infections, being characterized by a more complete immune reconstitution and a rare production of immunological rejection by the host [79].

Finally, the BLT (bone marrow, liver, and thymus) model, obtained by irradiating NOD/SCID or other strains of immunodeficient mice with sublethal doses, transplanting human embryonic thymus and homologous embryonic liver tissue, and injecting human CD34+ HSCs via the tail vein. This model, even if more complex, sensitive to GVHD, and time-consuming than the others, supports better development of humanized hematopoietic and immune systems [78].

Although humanized mice are critical for preclinical studies of human diseases, they still face challenges and limitations, such as cross-reactivity between species and incomplete human hematopoietic and immune cell development, due to the differences between mice and humans in the growth factors and cytokines required. Recent advancements in genetic engineering and human cytokine delivery methods have enhanced the functionality of these models, with the aim of ensuring the maintenance of human cells in the murine microenvironment [78].

Humanized in vivo models have also been employed for the study of primary bone malignancies. For instance, Ko et al. developed a humanized osteosarcoma mouse model injecting luciferase-expressing KHOS/NP cells at different time points in humanized NGS mice, performed by injection with human CD34+ hematopoietic stem cells, in order to study the influence of human cell engraftment osteosarcoma growth and progression [83]. Moreover, Wagner et al. reported a novel orthotopic humanized mouse model of osteosarcoma, implanting tissue engineered bone constructs seeded with human osteoblasts at the femur of NSG mice, subsequently bone marrow was transplanted with human CD34+ cells and orthotopically injected with Luc-SAOS-2 cells [84]. Interestingly, with the aim to investigate the effect of immunotherapy on osteosarcoma, Zheng et al. showed that nivolumab inhibited osteosarcoma metastasis to the lung in humanized PBMC-engrafted mice injected with KHOS cells, promoting CD4+ and CD8+ lymphocyte infiltration and increasing the cytolytic activity of CD8 lymphocytes in the lungs, but did not affect the growth of the primary tumor, suggesting that the efficiency of immunotherapy may be organ specific [85]. In the case of Ewing sarcoma, as already mentioned in Section 2.1.2, Luo et al. developed an orthotopic humanized mouse model of Ewing sarcoma, by transplanting fresh human cord blood CD34+ HSC into NSG-SGM3 mice combined with subsequent Ewing sarcoma patient-derived cell engraftment in the tibia of the humanized mice [67].

#### 2.1.3. Genetically Engineered Models

Genetically engineered mouse models allow researchers to manipulate specific genes known to be implicated in the development and progression of bone malignancies, mirroring genetic alterations observed in human cancer. These models are helpful to shed light into the molecular mechanisms and pathways driving bone sarcomas, including genetic initiator events, and facilitate preclinical testing of targeted therapies.

Especially regarding osteosarcoma, due to its high genetic heterogeneity, conditional activation of oncogenes or deletion of several suppressor genes known to be implicated in tumor initiation and progression, such as p53, RB, p14 alternative reading frame (p14ARF), p16INK4a, neurofibromatosis type 2 (NF2), p27, protein kinase cAMP-dependent type I regulatory subunit alpha (PRKAR1A), and p21CIP, is a common method used to construct transgenic mouse models. Other mutated genes implicated in osteosarcoma pathogenesis are c-FOS and TWIST. Among all of them, p53 and RB are the most frequent targets of silencing in conditional knock out mouse models [86,87]. Indeed, mice carrying p53 gene silencing specifically in osteoblast precursors develop osteosarcoma [88]. Interestingly, osteoblast-restricted co-deletion of RB and p53 can significantly accelerate cancer development in mice, which show the classical features of human OS, including cytogenetic and histological complexity and comparable gene expression signatures [89], while germline deletions of RB alone do not develop osteosarcoma [90]. Moreover, double-transgenic mice overexpressing c-Fos and c-Jun frequently develop osteosarcoma [91]. As metastasis is a major cause of cancer-related death in humans, it is important to consider the ability of transgenic models to recapitulate the metastatic cascade, which often varies by genotype.

Regarding Ewing sarcoma, instead, generation of transgenic mouse models turned out to be quite difficult. Several approaches have been tested, with poor results until the importance of selecting cells that tolerate EWRS1–FLI1 expression was demonstrated, since overexpression of the oncogenic fusion protein induces apoptosis in normal cells [92]. In 2021, Tanaka and Nakamura successfully described the EWSR1-FLI1-expressing mouse model of Ewing sarcoma by selecting chondrogenic progenitor cells, mouse embryonic superficial zone (eSZ) cells purified from embryos, transfecting with EWSR1–FLI1 plasmid, and transplanting them into Balb/c mice [93].

#### 2.1.4. Chemically Induced Models

Direct exposure of rodents to chemical carcinogens can be used to develop induced primary bone tumor models, which are mainly used to investigate environmental factors contributing to bone cancer initiation and to test chemo-preventive strategies. For example, initial in vivo studies revealed that experimental induction of osteosarcoma can happen through rat injection or irradiation with radionuclides, such as phosphorus-32 (^32^P), Americium-241 (^241^Am), Plutonium-239 (^239^P), or Cerium-144 (^144^Ce) [94,95,96]. Although the high penetrance of these models and the fact that they yield tumors that histologically resemble human cancer, their relevance is low, being representative of a therapy-induced disease, thus differing from the majority of human sarcomas that are sporadic. Other chemical agents that have been used to induce bone sarcomas by injection into rodents’ muscles include 12-dimethylbenzanthracene (DMBA), arsenate, beryllium zinc silicate, aflatoxin B1, and diethyl nitrosamine [97,98].

Despite being the first induced animal models, they are no longer used in cancer research, not only because they are not very reproducible, but for safety reasons in laboratories, given that chemical substances can be harmful to researchers. Finally, this procedure is classified as severe.

### 2.2. Animal Models of Breast Cancer-Induced Bone Metastases

Metastatic cells originating from advanced breast cancer frequently spread to bone as a secondary site in patients, leading to debilitating skeletal complications. Given the limited availability and difficulty of obtaining human bone metastatic samples, especially during oncological treatments, animal models have become instrumental in elucidating the complex interactions between breast cancer cells (seed) and the bone microenvironment (soil). These in vivo models allow researchers to investigate tumor cell homing, the molecular pathways behind the establishment of metastases, and subsequent bone destruction, simulating the multistep process of metastatic colonization generally found in human bone metastatic disease. They include the following: (1) transplantation of breast cancer cells into the systemic circulation (intracardiac and caudal injections), directly into the bone tissue (intraosseous injection) or into the organ of the primary tumor, specifically into the mammary fat pad (orthotopic injection), both for allograft and xenograft models; (2) transgenic mouse models (Table 5 and Table 6) [99,100]. Of note, spontaneous models of bone metastasis are very rare, as rodents and dogs develop mammary carcinomas which, however, do not normally invade the bone.

To date, mice have been the most common host used in preclinical studies of breast cancer-derived bone metastases, followed by rats. Zebrafish have also been used as a promising xenograft tumor model for preclinical studies on bone metastases [101,102].

#### 2.2.1. Breast Cancer Cell Line Injection or Tumor Fragments Implantation Models: Syngeneic and Xenograft Models

As for osteosarcoma models, the most widely used animal models for in vivo studies of bone metastasis are xenograft models, which involve injection of human cancer cell lines in immunocompromised mice or rats. Syngeneic models of mammary carcinoma are also used, especially if the intention is to investigate the important role of the immune system, preceded by an accurate selection of the primary cancer cell line to be implanted in an immunocompetent host. In the case of breast cancer cells, the human triple negative and highly osteotropic MDA-MB-231 cell line is likely the most employed, followed by the human T47D mammary ductal carcinoma cell line, while the epithelial like MCF7 cell line from mammary adenocarcinoma has a low ability for growth since these cells still express the estrogen receptor (ER); to foster in vivo growth animals are implanted with a 60-day slow release 17β-estradiol pellet (0.5 mg) before inoculation [47].

The murine 4T1 and E0771 cell lines from Balb/c and C57BL/6 mouse strains, respectively, grow quite fast when injected and show a preference for metastasis in the femur and tibia. Furthermore, to increase the frequency of bone metastases, both human and murine breast cancer cell sublines are now available, which have been established via repeated steps of injection of parental breast cancer cells and recovery of metastatic cells from bone lesions. In this way, subclones from human MDA-MB-231 cells with selective bone tropism have been produced, such as MDA-MB-231-B02, MDA-MB-231-B, MDA-MB-231-bone, and MDA-IV; these cells have shown a higher propensity to form rapidly growing osteolytic bone metastases compared to the parental cell line [103,104]. Consequently, mice developing visceral and/or bone metastases usually present with problems of locomotion, and experience pain and cachexia, therefore their monitoring is crucial to put in place the appropriate countermeasures, such as analgesia or euthanasia. For all these reasons, the classification of the procedure is severe.

##### Intravascular Injection

Tumor cell injection into systemic circulation allows investigation of most of the phases of the metastatic cascade, from survival within the bloodstream to eventual extravasation, invasion, homing, and growth of metastatic cancer cells into the bone compartment, relatively mimicking the human condition. Thus, they are one of the most used models for generating bone metastases for preclinical in vivo studies.

Intracardiac injection is performed by inoculation of breast cancer cells directly into the left ventricle, bypassing the lung vasculature, and often results in a widespread whole body metastasis formation, initially in the metaphysis of long bones (but also the spine and jaw can be affected), and then in visceral metastases, particularly in the lungs and lymph nodes [105,106]. The employment of bioluminescence imaging immediately after intracardiac injection is helpful to confirm a successful injection, before cancer cells circulate into the whole body. This technique allows researchers to perform a detailed study of breast cancer cell bone colonization and lodging in the bone metastatic niche, following over time their localization from the initial steps of invasion and bone colonization/homing to advanced stages of local bone disease, within about the first 3–4 weeks following inoculation [107]. The percentage of mice that develop metastases after intracardiac injection, as well as the affected sites, dimension, and number of metastases, are unpredictable, show considerable variability, and depend on the type of cancer cell line and mouse strain used. Usually between 50% and 70% of mice subjected to intracardiac injection develop mono or bilateral osteolytic lesions in both tibia and femurs, while the incidence of mortality strictly related to the site of injection is around 10%.

To date, the intracardiac model is a well-established and common technique employed to generate and study bone metastases. It has been extensively demonstrated that the intracardiac injection of human breast cancer cell lines, like MDA-MB-231, more aggressive and highly metastatic to bone, and MCF7, which displays low metastatic potential and long latency, in 4–6-week-old female immunodeficient mice results in bone metastases development, causing osteolytic lesions within 3–4 weeks in the case of MDA-MB-231; while mixed lesions (predominantly osteosclerotic) are obtained within 20–25 weeks in the case of MCF7, or within 10–12 weeks in the case of MCF7 stably transfected with the oncogene Neu (MCF7/Neu) in immunocompromised mice [108]. As already stated, the development of bone metastases after MCF7 cell intracardiac inoculation usually requires estradiol administration, which promotes in vivo growth of these ER positive cells [47].

Tail artery injection of rodents is easier to handle and offers better accuracy due to the visibility of caudal vessels. In this regard, their dilation prior to injection or the use of fluorescein to reveal vessel flow can make the injection easier, allowing them to obtain a higher rate of bone metastasis, with a very low incidence of metastases in visceral organs [109,110].

##### Intraosseous Injection

Inoculation of breast cancer cells directly into the proximal tibia or distal femur of mice has the potential to develop an efficient and easily reproducible model of osteolytic mammary tumor in bone, normally used to examine the growth and behavior of the primary tumor within the bone microenvironment. Both immunodeficient and immunocompetent animals are used, characterized by limited morbidity [111]. Compared to intravascular injections, it is less impactful for mice, in terms of mortality after injection (almost 0%) and the possibility to develop cachexia, although they can experience pain and reduced mobility. This model is useful to assess the molecular pathways involved in bone colonization and to test potential anti–metastatic and antiresorptive therapies [112,113]. However, it cannot be considered a real model of bone metastasis, as it bypasses all the early and most important stages of the metastatic cascade, reproducing only the growth in the host tissue, the osteolytic feature, and the tumor cell interaction with bone resident cells.

Regarding alternative intraosseous methods of inoculation, the E0771 luminal B mammary cancer cell line isolated from a spontaneous tumor in C57BL/6 mouse, has been recently used to model local bone metastasis of breast cancer through an intrafemoral inoculation of syngeneic mice in studies aimed at evaluating anti-metastatic treatments [114,115].

##### Orthotopic Injection

A more comprehensive in vivo model of bone metastasis, covering all the processes typical of human breast cancer, from the early stages of primary tumor growth to cell intravasation and metastatic spread to bone or other secondary sites, is represented by the orthotopic injection of breast cancer cells into their primary site, that is mammary gland fat pad both in mice and rats. Usually, the fourth mammary gland fat pad is preferred for injection (monolateral inoculation) since the first and the last should be avoided because it could hamper movement [52]. This model is known to produce low rates of mortality in mice (since it does not necessarily require the incision of cutting the teat, while it is suggested that a stereoscope is employed) and high penetrance of the tumor but does not show a high rate of bone metastasis and takes considerable time (usually 8–12 weeks) to generate them as compared to the high growth rate of the primary tumor which, as for the subcutaneous injection, should not exceed a tumor volume of 2 cm^3^. Alternatively, it is possible to surgically remove the primary tumor and leave the animal alive for a longer time, waiting for the development of metastases. Orthotopic injection results to be appropriate to study the in situ tumor growth, to estimate metastatic rate of different breast cancer cell lines, and to obtain preclinical information, like the evaluation of the potential therapeutic effects of biologically active molecules for the treatment of osteolytic bone metastases and induced bone loss.

The syngeneic model more accurately mimic metastatic cancer development observed, however mouse mammary cancer cells preferentially metastasize to lungs, while human breast cancer primarily metastasizes to bone. As an example, orthotopic injection of 4T1 cells in BALB/c mice induces visceral and bone metastases after the second week from the inoculation [116,117]. Over the years, several syngeneic models have been developed, also through the isolation of tumor cell sublines with higher bone–metastatic tendencies, via repeated in vivo passaging, to increase the success rate of bone metastasis [118]. Due to the possibility, although not so frequent, to induce metastases, this procedure is classified as severe.

#### 2.2.2. Genetically Engineered Models

In vivo studies of mammary carcinoma in genetically modified mice have shown morphogenic similarities to human disease. Among the many transgenic models developed over the years, it is important to consider the MMTV (mouse mammary tumor virus)-LTR-driven transgenic mouse model, which bears expression of oncogenes like c-Myc, neu/ErbB2, p53, Wnt1, and SV40-T-antigen under the control of the tissue selective MMTV-LTR promoter targeted to the mammary gland [119,120,121,122]. These models result in the development of breast cancer, with characteristic phenotypes, followed by lung or lymph node metastases, while those in the bone are rare. The low incidence of bone metastases is one of the major disadvantages of these models, in contrast to the rapid progression of the primary tumor. Thus, they are not appropriate models for investigating the molecular basis of breast cancer-induced bone metastasis or evaluating antimetastatic therapies, but they have proven to be important tools for studying genes or molecular drivers involved in primary breast cancer development and progression, as well as cancer prevention and therapeutic resistance.

### 2.3. Analysis of Animal Models of Primary and Secondary Bone Tumors

In the contest of in vivo cancer research, “in itinere” and “post mortem” analyses of animal models are crucial to characterize the development of primary bone tumors and breast cancer-derived skeletal metastases.

#### 2.3.1. In Itinere Analyses and Monitoring Animal Welfare

In vivo or in itinere analyses refer to the longitudinal monitoring and assessment of animal welfare as well as of tumor location, growth rate, presence and number of metastases in living animal models. Indeed, this type of analysis is critical for understanding dynamic biological processes and responses as they occur, utilizing various techniques including the following: (i) real-time imaging techniques, such as bioluminescence, in vivo microcomputed tomography (microCT), and high-resolution radiography; (ii) determination of tumor and/or bone biomarkers in blood, serum, or urine; (iii) health and disease parameters monitoring, such as body temperature, weight, symptoms of cachexia and hind limb motility [123]. In the case of subcutaneous/orthotopic injections or whenever the tumor grows outwards, it is possible to evaluate the grow rate by determining tumor volume with a caliper [52,99,111,112]. Briefly, the tumor is approximated to an ellipse, whose volume is determined by the $\frac{4}{3}$πabc formula, where a is the longest radius, b is the shortest radius and c is the depth of the tumor. Usually, this evaluation can be assessed twice a week. This analysis is well tolerated, simple, non-invasive and does not require expensive equipment; however, it gives an indication of the tumor size, which also includes fibrotic and necrotic tissue, and therefore is not able to discriminate between living and necrotic tumor cells.

Another complication that can be found for a subcutaneous injection is the development of ulcerations, which require the use of wound healing gel or cream or, if the ulceration worsens, to euthanasia.

Whatever the type of inoculation, the location and growth of the tumor can be better monitored over time via fluorescence or via bioluminescence, a high sensitivity in vivo bioimaging technique used for detection and quantification of tumor cell presence, proliferation, and time of appearance of micro or overt metastases in distant organs, including bone. Both are made possible through the injection of breast cancer cell lines previously transfected to stably express green fluorescent protein (GFP) and the firefly luciferase (Luc), respectively [123]. The latter, in the presence of luciferin systemically injected into anesthetized animals at the time of monitoring, converts this substrate into a bioluminescent product [111]. Only the light emitted from the viable Luc-labeled cells in living tissues is then appropriately recorded and quantified by the bioilluminator available in the laboratory.

In the case of bone lesions, high-resolution radiography has been widely used in small animals. Using a cabinet X-ray machine researchers can weekly detect large osteolytic/osteosclerotic lesions and area of abnormal bone remodeling in a rapid way; however, this technique does not allow us to identify micro metastatic lesions. The site of abnormal bone remodeling and metastases can be assessed also by microCT, an in vivo imaging option that allows for the acquisition and analysis at a high degree of spatial resolution of the 3D structure of bone [111]. It can provide both qualitative as well as quantitative analysis of bone structure, giving important measurements, such as trabecular number and thickness, trabecular separation, and cortical or trabecular bone volume per tissue volume [124]. Both these techniques provide information on cancer-derived bone lesions, but they do not provide direct information on tumor growth and distribution, as bioluminescence does.

To measure biochemical markers indicative of physiological states or responses to therapies, blood and urine can be regularly sampled during an in vivo experiment.

During an in vivo experiment animal welfare should be guaranteed by constant monitoring of animal health and behavior, to be carried out once or twice a day. For this reason, we can refer to the welfare guidelines proposed by Morton and Griffith in 1985 [125] and, more recently, to the OBSERVE guidelines [52]. In particular, animal health should be followed by planning a comprehensive and specific set of monitoring sheets for different clinical signs, including the description of specific humane endpoints (HEPs). These are specific timepoints at which measures to alleviate pain and distress are taken, and ultimately offer criteria for animal euthanasia. Several clinical signs can be monitored in real time, such as food and water intake, body weight loss, sarcopenia, cachexia, reduced mobility, and other symptoms of animal distress like arching of the back and respiratory distress [111,113]. Cachexia is a paraneoplastic syndrome characterized by dramatic body weight loss due to muscle and adipose tissue loss and it is caused by catabolism factors produced by tumors. Humaine animal killing is necessary in this case.

Pain is another clinical sign that needs to be checked and can be counteracted by providing appropriate analgesia. Animals subjected to intraosseous or intracardiac injection can experience pain, which can be assessed by applying the grimace scale [126] and, more specifically, by means of tests, like the incapacitance tester and the spontaneous deambulation tests [127,128]. The former features two scales that are able to discriminate weight distribution between the two hindlimbs. In normal conditions, rodents will tend to distribute the weight evenly between the two limbs, but when one of the two limbs experiences bone pain (i.e., the one subjected to intraosseous injection), mice will relieve them from some of their body weight, reducing the percentage of weight borne by that limb. In another test, that can be named spontaneous deambulation test, mice are placed in a 45 × 45 × 45 cm arena, then the trajectory of the mouse is recorded and quantified over a specific timeframe (e.g., 10 min) to assess the distance the mouse is willing to walk voluntarily, without external stimulation. Mice experiencing bone pain will start showing a decrease in spontaneous ambulation.

#### 2.3.2. Post Mortem Analyses

Post mortem analyses include the following: (i) anatomical gross dissections; (ii) blood recovery for tumors and/or bone remodeling biomarkers; (iii) histopathological analysis and immunohistochemistry of explanted tumors, histomorphometry of bone segments; and (iv) imaging techniques on bones, such as microCT and X-ray analysis.

At the end of an in vivo experiment, the animal model should be examined to check eventual visceral metastatic foci. All relevant organs and visible metastases are collected, examined at a first sight in size and color, fixed in 10% buffered formalin for 24–48 h to be further analyzed via histology, or frozen at −80 °C to proceed subsequently with molecular analysis. About the bone samples, especially long bones and lumbar vertebrae, after their collection and before proceeding with histopathological analysis, they could be subjected to ex vivo microCT, to assess bone lesions and bone structure parameters. Then, bone samples can be decalcified and paraffin-embedded, to be sectioned and stained for immunohistochemistry, or can be embedded in resin like polymethyl methacrylate for histomorphometric analysis. In the first case, common staining like hematoxylin and eosin (H&E) allow the detection and measurements of the tumor burden or the number and area of metastatic foci into each section of bone or other internal organ analyzed, by image analysis software. Immunohistochemistry can also be performed on tumor samples for the detection of cancer related proteins of interest as well as for the typical tumor markers (i.e., cytokeratins, ki-67) [111,129]. Bone histomorphometry, instead, consists in the evaluation of bone cell parameters, which include osteoblast number and surface/bone surface in bone sections stained with toluidine blue, and osteoclast number and surface/bone surface on sections stained for tartrate-resistant acid phosphatase (TRAcP) activity, that is known to increase with cancer-induced osteolysis.

Finally, ELISA assay can be performed on serum collected from animal at the time of sacrifice, to check for the presence of bone turnover markers, such as carboxy-terminal cross-linking telopeptide of type 1 collagen (CTX), TRAcP, bone alkaline phosphatase (BALP), and osteocalcin (OCN), hormones, inflammatory or tumor-derived growth factors [111].

### 2.4. The Era of Zebrafish: A Suitable Xenograft Model

The fish, Osteichthyes, *Danio rerio* (i.e., zebrafish) has recently emerged as a suitable model for studying developmental processes and human diseases, including cancer.

Zebrafish shares a high level of genetic and physiological homology with humans, requires low costs of maintenance and is quite prolific, being able to give over 100 embryos per clutch and, looking at an ethical point of view, is less sentient than other vertebrates [130]. Moreover, the adaptive immune system in zebrafish is not completely developed until 14-days post fertilization (DPF), which allows survival and metastatization of xenotransplanted human tumor cells [131]. It is possible to circumvent immune rejection of xenoimplant in older zebrafish by chemical immunosuppression [132] irradiation [133] or employing immunocompromised lines [134,135].

An additional advantage of this model is that zebrafish embryo and larvae are transparent, therefore a clear non-invasive time-lapse can be easily monitored over time. Moreover, a transparent adult zebrafish model, named Casper, has been developed and employed for tracking fluorescent or bioluminescent transplanted cells, including tumor cells [136]. As an example, injection of red-fluorescent tumor cells into the Tg(fli1: EGFP) transgenic zebrafish, in which vascular endothelial cells are labeled by green fluorescent protein, allowed to investigate both the process of tumor cell metastasis and changes in the vascular system throughout the body, by epifluorescence or confocal microscopy [137]. Moreover, engineered MDA-MB-231 fluorescent cells have been injected into the duct of Cuvier of the fli1:GFP transgenic zebrafish embryo and within 6 days these cells circulated and reached the tail fin forming micrometastases [138].

Other breast cancer cell lines have been successfully injected into the perivitelline space or into the Cuvier duct of embryo zebrafish to observe the development of metastases, such MCF10A and the MF10Aras subclone [139].

Zebrafish have also been employed to model primary bone tumors [140]. The Ewing sarcoma cell line TC32 has been injected into the yolk sac of Casper embryos and after 24–120 h of injection these cells were able to migrate into the tail, by a mechanism dependent on Y-box binding protein 1 (YB-1), via HIF1α expression [141]. Similarly, van der Ent et al. employed a zebrafish model to inhibit Ewing sarcoma by disruption of EWSR1-FLI1 transcriptional activity and reactivate of p53 [142].

Different studies have adopted the zebrafish model to model osteosarcoma. Injection of the MG-63 cell line allowed tests of the antineoplastic and anti-angiogenic action of noscapine [143], of nimbin [144], and of natural pigments [145]. Other osteosarcoma cell lines successfully injected into zebrafish to study tumor extravasation and metastasis include U2-OS, SaoS2, HOS, of human origin [146,147], as well as a canine osteosarcoma cell line [148].

## 3. Organoids and Organ-on-Chip: Towards a Total Replacement?

Although it is well recognized the fundamental contribution that the in vivo experimentation gives, the scientific community is being even more conscious of the need to identify new strategies able to reduce the use of animals.

### Organoids and Organ-on-a-Chip

All the animal models previously described have been widely used to study primary and secondary bone tumors. However, the need to find alternative methods remains the main challenge nowadays, in accordance with the 3R’s principle. In recent years, significant advancements have been made to achieve this goal, leading to alternative solutions such as organoids and organ-on-a-chip technologies, with related benefits such as cost-effectiveness, time efficiency, less complex testing procedures, and societal benefits [149].

Organoids are structures derived from self-organizing cells in 3D cultures, characterized by organ-specific features. They are advantageous over traditional 2D cell cultures, as they can show near-physiological cellular composition and actions [150]. Currently, the use of organoids in the study of both primary and secondary bone tumors is increasing. For instance, Dorn et al. recently demonstrated the possibility of using osteosarcoma human biopsies and cell lines such as SaOS-2 and MG-63 to produce organoids in a 3D in vivo chorioallantoic membrane (CAM) model [151]; while He et al. first reported an organoid culture system derived from primary or lung metastatic osteosarcoma specimens [152]. Moreover, Ding et al. subjected breast cancer–bone metastases patient-derived organoids to bulk DNA/RNA and single-cell RNA sequencing (scRNAseq), revealing intratumor heterogeneity and evolution, and potential therapeutic targets for precision medicine [153].

Currently, technology development of 3D bioprinting organoids is underway, promising better productivity. This includes inkjet-based bioprinting, laser-assisted bioprinting, extrusion-based bioprinting, and photo-curing bioprinting [154]. Despite these new technological improvements, organoids still have limitations that make them unsuitable for the full replacement of animal models. Organoids lack vasculature structures, which affect their growth and maturation, leading to differences in behavior compared to the original tissue [155]. Organoids also lack connections with neighboring tissues, commonly seen in living organisms, which is crucial when assessing metabolic health, making it difficult to create treatments for diseases. Additionally, organoids lack several cell types, structural organization, and physiological functions compared to functioning organs, limiting their ability to accurately replicate diseases and responses to treatment [156].

Organ-on-a-chip technology can be considered a merge between biology and microtechnology, as it represents microfluidic cell culture devices [157]. Organ-on-a-chip improves on the well-established alternative method of organoids, as this type of system allows cell communication between neighboring tissues. Chips are generally designed by collecting cells (primary cells, transformed cell lines, human ESCs, or iPSCs) using equipment with pumps (that enable fluid flow), incubators, sensors, and microscopes to monitor and examine the cells in the system [157]. Moreover, cells can be aggregated in matrix- or matrix-free conditions, depending on the cell type and physiological conditions [157]. Tumor-on-a-chip models have been developed to recreate and study tumor physiology and pathological functions in vitro [158]. For instance, Lu et al. recently developed an osteosarcoma-on-a-chip model to investigate osteosarcoma matrix-cell interactions and drug response [159]. The system is complex and includes a decellularized osteosarcoma extracellular matrix (dOsEM) with a fibrin (dOsEM-fibrin) hydrogel loaded with hematopoietic bone marrow mesenchymal stem cell (hBMSC)-derived extracellular vesicles (EVs), as an acellular bioink (dOsEM-EVs) to accurately replicate the physiochemical characteristics of the OS microenvironment. A 3D micro-osteosarcoma (micro-OS) was further constructed with dOsEM-EVs through 3D printing to recapitulate the spatial structure and cell distribution of OS. Then, the micro-OS combined with a microfluidic system was integrated into a multistage biomimetic osteosarcoma-on-a-chip (OOC) with a built-in recirculating perfusion system to test the drug screening potential, thus recreating an in vitro model of osteosarcoma [159]. As for secondary bone tumors, Hao et al. generated a spontaneous 3D bone-on-a-chip for the study of bone metastasis of breast cancer [160]. In this system, the chip is based on an osteoblastic tissue of up to 85 μm thickness, containing heavily mineralized collagen fibers naturally formed in 720 h without the aid of differentiation agents. Researchers then made co-cultures of the metastatic human breast cancer cell line MDA-MB-231 with the osteoblastic tissue developed in the chip, recreating an in vitro model of breast cancer bone metastases [160]. However, a limitation of the organ-on-a-chip is its complex experimental setup, which can be mitigated with clear guidelines or protocols.

In conclusion, despite these steps forward, organoids and organs-on-a-chip are currently unable to completely replace the use of animal models, serving instead as partial alternatives. The reason is related to the extreme complexity of the physiology and pathophysiology of organisms, which cannot be fully simulated with these in vitro models. However, considering the ongoing technological evolution, in the coming years we will likely see a gradual reduction in the use of animal models (which has already begun thanks to the previously described systems) and, hopefully, a complete replacement with better alternative methods.

## Figures and Tables

**Table 1:** Spontaneous animal models of osteosarcoma.

Animal Model	Species	Genetic Mutations	Pros	Cons
Spontaneous	Canine	p53, RB	Histopathologically and genetically similar to the human disease	- Heterogenic tumor - Different age of tumor diagnosis/development - Treatment decision dependent on the owner

RB = retinoblastoma.

**Table 2:** Heterotopic and orthotopic injection of osteosarcoma cells.

Inoculation Method (Allograft/Xenograft)	Species	Mortality Due to Inoculation	OS Growth and Incidence	Lung Metastases	Pros	Cons
Subcutaneous	Mouse	0%	High (100%)	NO	- Easy inoculation - Easily accessible tumor - No cachexia - Severity assessment: mild/moderate	- Not reflecting the natural tumor microenvironment - No metastases - Possible ulceration
Intravenous (tail vein)	Mouse	Low	–	YES	Study of tumor extravasation and lung metastases development	- The inoculation requires technical skill - Not reflecting prerequisite steps of metastasis - Possible development of cachexia - Severity assessment: severe
Orthotopic (Paratibial/Intratibial)	Mouse	0%–rare	High (90%)	YES	- Most used to study primary tumor growth, invasion and metastases - High metastasis rate	- The inoculation requires technical skill - Possible development of cachexia - Severity assessment: severe

OS = Osteosarcoma.

**Table 3:** Genetically engineered animal models of primary bone tumors.

Animal Model	Bone Tumor	Species	Genetic Mutations	Transgenic Models	Pros	Cons
Genetically engineered	OS	Mouse	Frequent: p53, RB Others: p14ARF, p16, p21, p27, NF2, PRKAR1A, c-FOS, TWIST	Conditional knock out	- Study of genetic initiators and molecular mechanisms driving bone sarcoma progression - Mirror genetic alterations observed in human disease	- Expensive
	ES	Mouse	EWRS1-FLI1	Conditional expression	Mirror genetic alterations observed in human disease	- Difficult to generate - Expensive - Strain difference in the tumor susceptibility

OS = Osteosarcoma; ES = Ewing sarcoma; RB = Retinoblastoma; ARF = Alternate Reading Frame; NF2 = Neurofibromatosis type 2; PRKAR1A = Protein kinase cAMP-dependent type I regulatory subunit alpha.

**Table 4:** Chemically induced animal models of osteosarcoma.

Animal Model	Bone Tumor	Species	Inducer	Pros	Cons
Chemically induced	OS	Rat	^32^P, ^241^Am, ^239^Pu, ^144^Ce	- Study of environmental factors contributing to primary tumor initiation - Test of chemo-preventive strategies	- Low relevance - Low reproducibility - Harmful for the operators - Severity assessment: severe

OS = Osteosarcoma; ^32^P = Phosphorus-32; ^241^Am = Americium-241; ^239^Pu = Plutonium-239; ^144^Ce = Cerium-144.

**Table 5:** Heterotopic and orthotopic injection of breast cancer cell lines.

Inoculation Method (Syngeneic/Xenograft)	Species	Mortality Due to Inoculation	Osteolytic Lesions Incidence	Pros	Cons
Intracardiac	Mouse	10%	50–70%	- Easily producing metastases - Recapitulate most of the steps of the metastatic cascade	- The inoculation requires technical skills - Possible development of visceral metastases - High variability - Development of pain, cachexia and problems of deambulation - Severity assessment: severe
Intravenous (caudal artery)	Mouse	0%-rare	High (70%)	- Low incidence of metastases in visceral organs - High accuracy for visualization of vessels - Widespread whole body - metastasis formation	- The inoculation requires technical skills - Development of pain, cachexia and problems of deambulation - Severity assessment: severe
Intraosseous	Mouse	0%	100%	Suitable model to study the ability of breast cancer cells to growth within the bone	- Not reflecting a real model of bone metastases, bypassing most of the stages - Problems of pain and deambulation - Severity assessment: severe
Orthotopic (mammary gland fat pad)	Mouse	0%	Low (10–20%)	Complete model to study primary tumor growth in situ and spread to distant organs	- Low bone metastases success rate - Development of visceral metastases - Possible development of pain, cachexia and problems of deambulation - Severity assessment: severe

**Table 6:** Genetically engineered animal models of breast cancer-induced bone metastases.

Animal Model	Species	Genetic Mutations	Pros	Cons
Genetically engineered	Mouse	c-Myc, neu/ErbB2, p53, Wnt1, and SV40-T-antigen under the control of the tissue selective MMTV-LTR promoter	- Morphogenic similarities to breast cancer human disease - Used to study genes or molecular drivers involved in primary breast cancer development and progression	- Rare incidence of bone metastases - Not reflecting an appropriate model to study bone metastases

## Data Availability

Not applicable.
